# Bystander CD4 T-cell death is inhibited by broadly neutralizing anti-HIV antibodies only at levels blocking cell-to-cell viral transmission

**DOI:** 10.1016/j.jbc.2021.101098

**Published:** 2021-08-19

**Authors:** Xiaoyu Luo, Hugo Mouquet, Olivier Schwartz, Warner C. Greene

**Affiliations:** 1 J. David Gladstone Institutes of Virology, San Francisco, California, USA; 2 Laboratory of Humoral Immunology, Department of Immunology, Institut Pasteur, INSERM U1222, Paris, France; 3 Virus and Immunity Unit, Department of Virology, Institut Pasteur, Paris, France; 4 Department of Virology, CNRS-UMR3569, Paris, France; 5 Basic Division, Vaccine Research Institute, Créteil, France; 6 Department of Medicine, University of California San Francisco, San Francisco, California, USA; 7 Department of Microbiology & Immunology, University of California San Francisco, San Francisco, California, USA

**Keywords:** HIV, cell death, infection, cell–cell interaction, apoptosis, pyroptosis, broadly neutralizing anti-HIV antibodies, cell-free virus transmission, bNAb, broadly neutralizing anti-HIV antibody, CMAC, 7-amino-4-chloromethylcoumarin, FACS, fluorescence-activated cell sorting, HLAC, human lymphoid aggregate culture, IgG, immunoglobulin G

## Abstract

The progressive loss of CD4+ T cells during HIV infection of lymphoid tissues involves both the apoptotic death of activated and productively infected CD4 T cells and the pyroptotic death of large numbers of resting and abortively infected bystander CD4 T cells. HIV spreads both through cellular release of virions and cell-to-cell transmission involving the formation of virological synapses. Cell-to-cell transmission results in high-level transfer of large quantities of virions to the target cell exceeding that achieved with cell-free virions. Broadly neutralizing anti-HIV antibodies (bNAbs) binding to HIV envelope protein capably block cell-free virus spread, and when added at higher concentrations can also interdict cell-to-cell transmission. Exploiting these distinct dose–response differences, we now show that four different bNAbs block the pyroptotic death of bystander cells, but only when added at concentrations sufficient to block cell-to-cell transmission. These findings further support the conclusion that HIV killing of abortively infected bystander CD4 T cells requires cell-to-cell transfer of virions. As bNAbs attract more interest as potential therapeutics, it will be important to consider the higher concentrations of these antibodies required to block the inflammatory death of bystander CD4 T cells.

In the absence of treatment, HIV infection leads to the progressive depletion of CD4 T cells and emergence of the AIDS (1, 2, 3, 4). Activated CD4 T cells are highly permissive to HIV infection and support viral replication and spread before dying by caspase-3-dependent apoptosis (5, 6, 7, 8). However, the number of activated CD4 T cells is not sufficient to account for the massive CD4 cell losses that occur during untreated HIV infection. These findings prompted consideration of several different mechanisms producing the demise of uninfected bystander CD4 T cells (9, 10, 11, 12, 13, 14). We have described one mechanism of bystander cell death that occurs in lymphoid tissue, but not in blood, involving abortive infection of neighboring resting (nonpermissive) CD4 T cells. Incomplete reverse transcripts accumulate in these abortively infected cells and are detected by the IFI16 DNA sensor triggering the activation of inflammasome assembly and death of cells by caspase-1/gasdermin D-dependent pyroptosis, a highly inflammatory form of programmed cell death (7, 15, 16, 17, 18).

During the HIV life cycle, cell-free viral particles bud from productively infected cells and initiate spreading infection through gp120-mediated binding to CD4 and subsequent engagement of the CCR5/CXCR4 chemokine coreceptors resulting in gp41-dependent virion fusion. Alternatively, virions can be transferred directly to neighboring cells through cell-to-cell contacts resulting in a 10- to 1000-fold more efficient transfer compared with infection with cell-free virions (19, 20, 21, 22, 23, 24, 25). Using a human lymphoid aggregate culture (HLAC) coculture system formed with cells from lymphoid tissue, we have suggested that abortive HIV infection and bystander cell pyroptosis require cell-to-cell virus transmission based on transwell experiments, antibody-mediated interruption of leukocyte function–associated antigen-1/intercellular adhesion molecule-1–dependent virological synapses, and alterations in surface area of cell cultures that favor or disfavor cell-to-cell interactions (26).

Anti-HIV-1 broadly neutralizing antibodies (bNAbs) are found in a small fraction of HIV-infected individuals (27). These antibodies neutralize a diverse range of HIV-1 viral strains by targeting multiple binding sites on HIV envelope protein (27). These bNAbs are being evaluated as therapeutics and also have been used to reprogram HIV-specific chimeric antigen receptor T cells (28, 29, 30, 31).

These bNAbs are capable of potently blocking infection by cell-free HIV virions (32, 33, 34). Other studies show that bNAbs also inhibit cell-to-cell virus transmission, but only when markedly greater amounts of antibody are added. Complete inhibition is sometimes not achieved (35, 36, 37). In the current study, we have analyzed the ability of four different potent bNAbs (NIH45–46, 3BNC117, VRC01, and 10E8) to block cell-free HIV transmission, cell-to-cell HIV transmission, and pyroptotic CD4 T-cell death in lymphoid tissues. The distinctly different dose–response profiles for bNAb inhibition of cell-free virion *versus* cell-to-cell transmission provided an independent method to test whether cell-to-cell transmission of HIV is required for the activation of the pyroptotic death pathway activated by abortive infection of bystander CD4 T cells.

## Results

**Figure 1:**
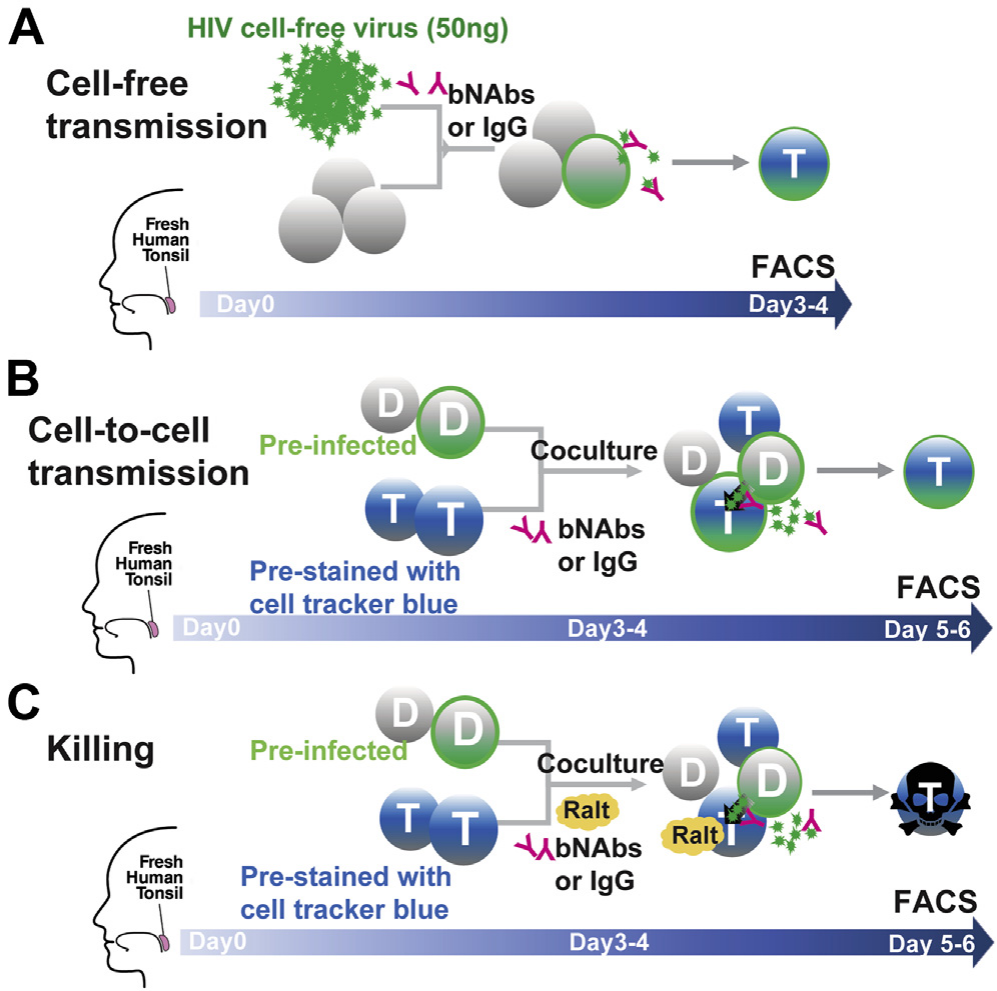
**Experimental design for measuring bNAb inhibition of cell-free HIV transmission, cell-to-cell virion transmission, and HIV-associated abortive infection and death of bystander CD4 T cells.***A*, HLAC cells were prepared using fresh human tonsil tissue on day 0, spinoculated with 50 ng of HIV.GFP preincubated with bNAbs, and then cultured together at 37 °C for 3 to 4 days before flow cytometric analysis. The detection of GFP+ cells in the bNAb-treated samples indicates the extent to which cell-free virus transmission escaped antibody neutralization. *B*, fresh tonsil cells were prepared and divided into donor (D) and target cell (T) populations on day 0. D cells were infected with HIV.GFP for 3 to 4 days when 1 to 2% of the CD4 T cells were productively infected. The T cells were stained with a CellTracker Blue preincubated with bNAbs over a range of different concentrations and cocultured with the infected donor cells. The coculture was continued in the presence of the bNAbs or control immunoglobulin G for 48 h to allow for spreading infection through direct cell-to-cell contact. Cultures were harvested, and cells were analyzed by flow cytometry to determine numbers of GFP+ target cells. The degree to which spreading infection occurred in the presence of either bNAbs or immunoglobulin G control was accessed at day 5 to 6. Note that a low level of *de novo* free virus (3–12 ng/well) was produced and released by the donor cells during the 48 h of coculture. *C*, to test the ability of bNAbs to inhibit cell death related to abortive HIV infection in bystander CD4 T cells, coculture experiments similar to those described in condition “*B*” were conducted with the addition of an integrase inhibitor, raltegravir (Ralt), before and during the coculture. Raltegravir was added to block spreading productive infection in the target cells. Importantly, this drug does not interfere with abortive infection and ensuing pyroptotic cell death since the drug acts after the generation of incomplete reverse transcripts and sensing of these viral DNAs by IFI16. Cells were collected on days 5 and 6 for flow cytometric analysis to assess levels of target cell depletion. bNAb, broadly neutralizing anti-HIV antibody; HLAC, human lymphoid aggregate culture.

**Figure 2:**
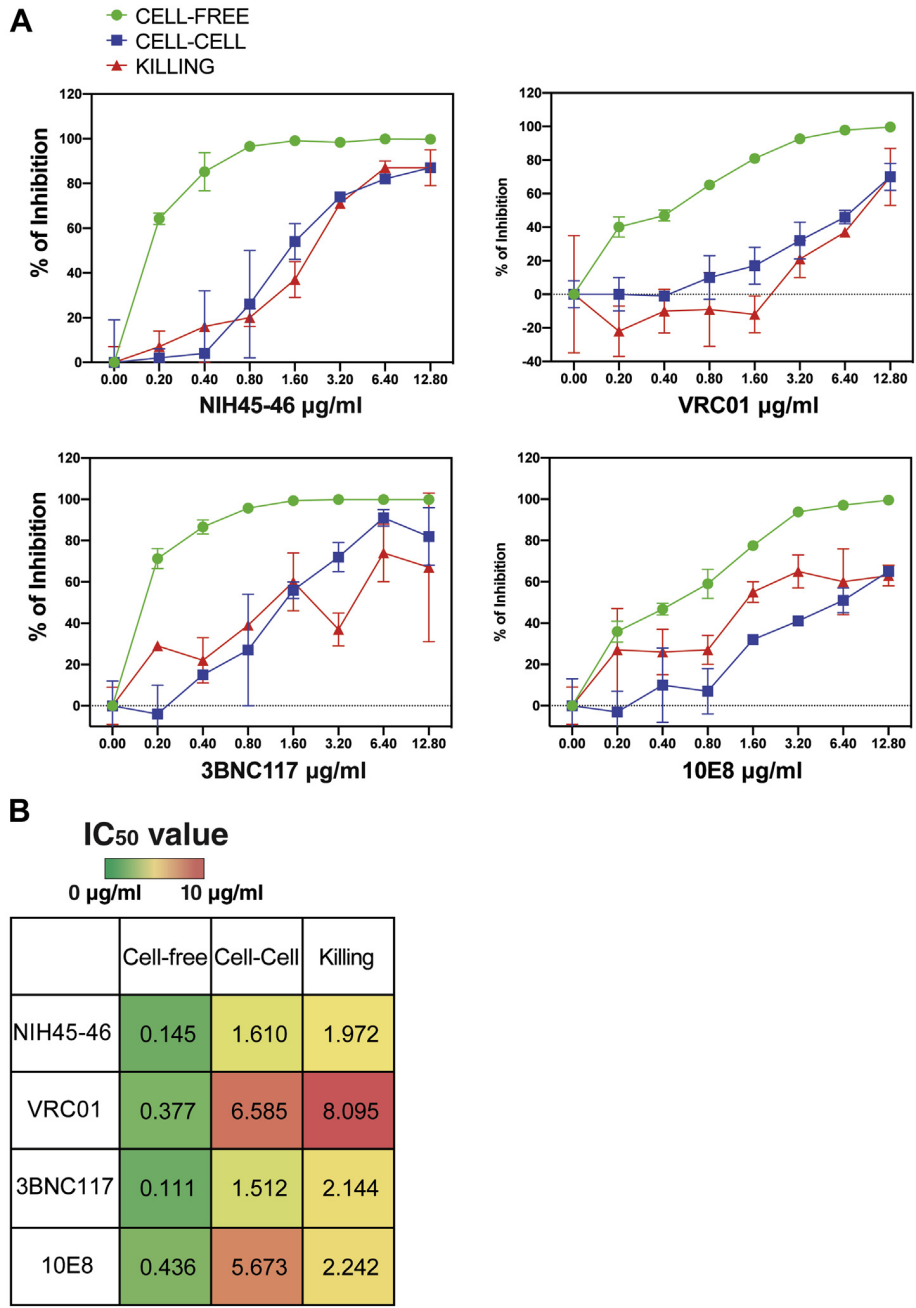
**Comparing the dose response for bNAb inhibition of cell-free virion transmission *versus* cell-to-cell transmission with the dose response required for inhibition of abortive HIV infection and bystander CD4 T-cell death.***A*, percentage of bNAb inhibition of cell-free virus transmission (*green lines*); cell-to-cell virus transmission (*blue lines*); and abortive infection/cell killing (*red lines*) were plotted as line graphs for four different bNAbs (NIH45–46; VRC01; 3BNC117; and 10E8) tested at eight different concentrations ranging from 0 to 12.8 μg/ml. Each data point was measured and calculated as described in Figure 1 and Experimental procedures section. The data represent mean + SD of triplicate samples. This experiment was repeated six times with similar results using tonsils from different donors. *B*, IC50 values (μg/ml) of neutralizing antibody (NIH45–46; VRC01; 3BNC117; and 10E8) inhibition of NLENG1-IRES-GFP (NL4-3)-cell-free virus transmission, cell–cell transmission, and CD4 T-cell killing. bNAb, broadly neutralizing anti-HIV antibody.

To compare the ability of bNAbs to inhibit cell-free, cell-to-cell, and HIV-associated bystander killing, different experiments were performed, each incorporating a broad range of bNAb concentrations (see Experimental procedures section) (Fig. 1). Consistent with prior reports (35, 38), we found that the NIH45–46 and 3BNC117 bNAbs efficiently blocked >80% of cell-free virus infection at a concentration of 0.4 μg/ml and reached close to 100% inhibition at a concentration of 0.8 μg/ml. Comparable inhibition by the VRC01 and 10E8 bNAbs generally required higher antibody concentrations (80% inhibition of cell-free virus infection at 1.6 μg/ml and 100% inhibition at 6.4–12.8 μg/ml) (Fig. 2*A*, *green lines*).

**Figure 3:**
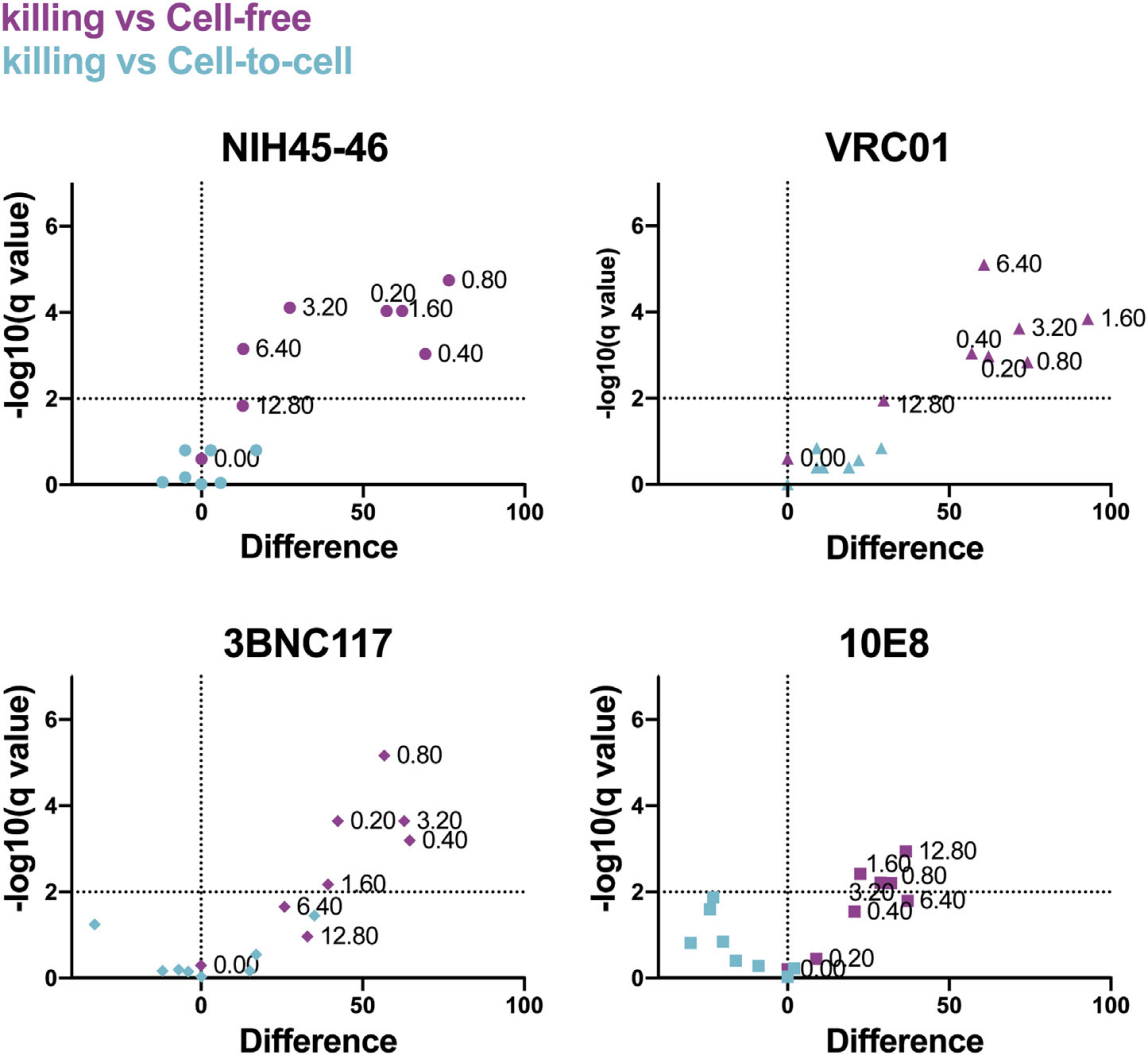
**Volcano plots of multiple *t* test show bNAb inhibition of cell-to-cell transmission, and bystander CD4 T-cell death is statistically indistinguishable.** Multiple *t* tests for each bNAb at each concentration were conducted, and the difference of mean (% of inhibition) for cell death *versus* cell-to-cell transmission (*cyan*) or cell death *versus* cell-free transmission (*purple*) was plotted on the *x*-axis in a volcano plot for each bNAb. *Dotted line* at *x* = 0 indicates no differences. −log (*q* values) are plotted on the *y*-axis. *q* values (false discovery rate–adjusted *p* values) >0.01 indicate that the differences observed are statistically significant (labeled by *dotted vertical line* at *y* = 2). Different bNAbs are plotted in separate graphs with different shapes. Concentrations (μg/ml) for each bNAb are annotated next to each data point. bNAb, broadly neutralizing anti-HIV antibody.

For each of the four bNAbs tested, the antibody concentration required to block cell-to-cell infection was more than 10 times higher than that required to block cell-free infection (Fig. 2*A*, *blue lines*). Of note, the level of inhibition of cell-to-cell virus transmission was highly correlated with the level of inhibition of killing of bystander CD4 T cells for each of the four tested bNAbs (Fig. 2*A*, *red lines*) although inhibition of cell killing by the 10E8 bNAb, uniquely targeting the membrane proximal external region, was not quite as effective as inhibition of cell-to-cell infection in the lower dose range. The IC_50_ for all four bNAbs in the context of cell-to-cell transmission and killing was approximately 10 times higher than that of cell-free transmission (Fig. 2*B*). Application of multiple *t* tests indicated that bNAb inhibition of cell-cell transmission and bNAb inhibition of killing were statistically indistinguishable (Fig. 3, *cyan*), whereas significant differences existed in the level of inhibition with an adjusted *p* value (*q*) >0.01 between cell killing and bNAb inhibition of cell-free viral infection (Fig. 3, *purple*) for each bNAb and concentration tested. All the aforementioned results confirmed the strong correlation between bNAb-mediated neutralization of cell-to-cell transmission and inhibition of abortive infection and depletion of bystander CD4 T cells.

One confounding aspect of this study was our inability to measure effects of the bNAbs on cell-to-cell transmission while not also inhibiting cell-free virus transmission at the same time. In the HLAC coculture experiment (Fig. 1, *B* and *C*), the infected donor cells in each well released ∼3 to 12 ng of cell-free virus particles during the 48 h of coculture as measured by ELISA. Of note, when these supernatants were collected and added to fresh tonsil cultures, little or no productive infection was observed and no CD4 T-cell killing was detected after 48 h (data not shown). These findings indicate that T-cell depletion occurred, although cell-free virion release although by donor cells during the 48 h coculture period was negligible even in the absence of bNAb addition.

Together, these results confirm that HIV-specific bNAbs are able to block cell-to-cell transmission provided these antibodies are added at sufficiently high concentrations to block this efficient pathway. We further find that these bNAbs block the death of abortively infected bystander CD4 T cells when added at the high concentrations needed to block cell-to-cell transmission but not at the lower concentrations blocking cell-free virus infection. These findings provide further support to a critical role for cell-to-cell transmission of virus leading to abortive infection of nonpermissive CD4 bystander T cells and their subsequent death by caspase-1–activated pyroptosis.

## Discussion

Like many mammalian viruses, HIV effectively spreads to new target CD4 T cells either through the release of cell-free virions or through cell-to-cell transfer of virions across virological synapses. Cell-to-cell transmission is a 10 to 1000 more efficient process for viral spread than infection by cell-free virions (19, 20, 21, 22, 23, 24, 25). These different routes of viral spread have been extensively studied in model cell lines and activated peripheral blood mononuclear cells that are highly permissive to HIV infection. However, less is known about the importance of these pathways in lymphoid tissue where most viral replication, spread, and cell death occurs.

Using tonsil or spleen tissue to prepare HLAC, we have identified a mechanism of HIV-associated CD4 T-cell depletion that occurs in lymphoid tissues but is not found in circulating blood CD4 T cells (17). Our findings indicate that this death pathway requires cell-to-cell transmission of HIV to neighboring nonpermissive (resting) CD4 T cells that become abortively infected. IFI16 sensing of the levels of HIV DNA produced by cell-to-cell spread triggers inflammasome assembly, caspase-1 activation, and the death of these cells by inflammatory pyroptosis (7, 15, 16). Disruption of virological synapses by addition of anti–leukocyte function–associated antigen-1 or anti–intercellular adhesion molecule-1 antibodies inhibits pyroptotic cell death, but these antibodies could also interfere cell-free virion infection because of virion incorporation of these plasma membrane proteins during budding (26). Increasing the surface area of cell culture, which decreases the opportunity for cell-to-cell transmission, also diminishes abortive infection and pyroptotic cell death (26). The requirement for cell-to-cell viral transmission may reflect the need for high-efficiency virion transfer that exceeds the threshold level of DNA transcripts needed to initiate IFI16 sensing.

The availability of multiple bNAbs that block both cell-free or by cell-to-cell virion transmission at distinctly different antibody concentrations offered an opportunity to further test the role of cell-to-cell virion transmission for abortive infection and bystander cell death by pyroptosis. Schwartz *et al.* (35) had identified a subset of bNAbs that efficiently block cell-to-cell HIV transmission. We evaluated four of these antibodies in the current study: NIH45–46, 3BNC117, VRC01 (targeting the CD4-binding site), and 10E8 (targeting the gp41 membrane-proximal external region). In agreement with their previous report, the NIH45–46 and 3BNC117 antibodies display the highest potency, whereas the VRC01 and 10E8 antibodies are less potent. All four of these antibodies block cell-free virus infection, albeit at different relative concentrations. Similarly, all four antibodies block cell-to-cell viral transmission but only when added at roughly 10- to 20-fold higher concentrations. Importantly, we find that all the four bNAbs also blocked bystander CD4 T-cell depletion but only when added at the concentrations needed to block cell-to-cell viral transmission. These findings confirm and extend the conclusion that cell-to-cell transmission of HIV virions play an important role in dramatic loss of bystander CD4 T cells occurring in lymphoid tissues during untreated infection.

Interest in the potential use of HIV bNAbs as therapeutics or prophylactic is also increasing (39). Our current study emphasizes how bNAbs can be employed to disrupt HIV-associated bystander CD4 T-cell death and its associated inflammation. However, to achieve these effects, our study suggests that higher antibody concentrations will be required compared with levels needed to block cell-free virion infection.

## Experimental procedures

### Cells and reagents

Human tonsils were obtained from the National Disease Research Interchange or the Cooperative Human Tissue Network during routine tonsillectomies mainly for sleep disorders. HLAC cells were isolated and cultured as previously described (16). Briefly, tonsil tissue was dissected and pressed through a 40-μm mesh to create a single-cell suspension. Live HLACs were isolated by Ficoll density gradient centrifugation. HLACs were cultured in tonsil culture media as previously described (16). bNAbs (NIH45–46, 3BNC117, VRC01, and 10E8) and an IgG control were provided by Dr Hugo Mouquet (Institut Pasteur, France). Phycoerythrin-conjugated mouse anti-CD4 antibody (340670), allophycocyanin-conjugated mouse anti-CD8 antibody (340584), and allophycocyanin-H7-conjugated mouse anti-CD3 antibody (560176) used for flow cytometry staining were purchased from BD Biosciences. Zombie Aqua fixable viability kits (423102) were purchased from BioLegend. CellTracker Blue 7-amino-4-chloromethylcoumarin (CMAC) dye (C2110) was purchased from Thermo Fisher Scientific. Raltegravir (sc-208296) was purchased from Santa Cruz Biotech.

### Virus preparation

Proviral expression vector DNA encoding pNLENG1-IRES-GFP reporter virus was transfected into 293T cells using the Promega Fugene HD transfection reagent (E2311) and cultured at 37 °C. Media were replaced after 16 h, and culture supernatants were collected at 24 and 48 h. Virions were concentrated by ultracentrifugation. HIV.GFP viral stocks were quantitated by measuring Gag-p24 levels by ELISA. The pNLENG1-IRES clone was derived from NL4-3 as previously described (40).

### Measuring bNAb inhibition of cell-free virus infection

HIV.GFP (50 ng of Gag-p24) was mixed with serial dilutions of either bNAbs or an immunoglobulin G (IgG) control antibody (concentrations: 0, 0.2, 0.4, 0.8, 1.6, 3.2, 6.4, and 12.8 μg/ml). After 1 h of incubation at 37 °C, these virus and antibody mixes were added to one million HLAC cells in 100 μl total volume in 96-well V-bottom plates. Cells were spinoculated with virus (2 h centrifugation [1200*g*] at 25 °C) and then cultured at 37 °C as a pellet and harvested for fluorescence-activated cell sorting (FACS) analysis of GFP+ CD4 T cells every 24 h for 3 to 4 days with monitoring of infection levels in the untreated controls (aiming for 1 to 2% of HIV.GFP+ in CD4 T cells (15)). Inhibition of cell-free virus transmission by each bNAb treatment was calculated as follows:

*% of inhibition of infection = (% of infection in untreated control CD4 T − % of infection in CD4 T with bNAb treatment)/% of infection in untreated control CD4 T × 100%*

The final result for each bNAb concentration was further normalized by subtracting the baseline level of inhibition obtained with the control IgG. For example:

*Final % of inhibition of infection (NIH45–46, 12.8 μg/ml) = % of inhibition of infection NIH45–46, 12.8 μg/ml − % of inhibition of infection (IgG, 12.8 μg/ml).*

Note that the IgG control never produced inhibitory effects exceeding 10% of that detected with the bNAbs even at the highest concentrations.

### HLAC coculture experiments

Inhibition of cell-to-cell virus transmission by the bNAbs was tested as illustrated in Figure 1*B*. Equal numbers of freshly isolated HLAC cells were separated into “donor” and “target” cell populations. One million donor cells were infected with HIV.GFP (50 ng Gag-p24) in 96-well V-bottom plates by spinoculation (100 μl total volume, 2000 rpm, 25 °C, and 2 h centrifugation). Donor cells were cultured until an adequate level of infection appeared in the untreated control, usually around day 3 to 4 (1–2% of HIV.GFP+ in CD4 T cells, monitored every 24 h by FACS). Next, target cells were prestained with CellTracker Blue CMAC dye before being mixed with donor cells in fresh media in the presence of the individual bNAbs or control IgG for an additional 48 h. Cells were harvested for FACS analysis of HIV.GFP+ cells in the CellTracker Blue CMAC-labeled CD4 T cells. Inhibition of cell-to-cell virus transmission by each bNAb treatment was calculated with the same equation stated previously for calculating the cell-free virus transmission except samples were pregated only on the target cell population.

To assess bNAb inhibition of HIV-associated bystander killing, it was necessary to inhibit the generation of new productively infected donor cells, which could artifactually increase levels of cell killing, but still allow virus transmission and abortive infection to occur.

Target cells were pretreated with the integrase inhibitor raltegravir (15 μM) to block new productive infection while not affecting HIV abortive infection and bystander cell death that depends on the generation and sensing of incomplete HIV reverse transcripts formed before viral integration. The coculture experiment described previously was also conducted in the presence of 15 μM raltegravir when infected donor and target cells were mixed. Cells were harvested for flow cytometric analysis to determine remaining live CD4 T and CD8 T cells in the culture. The inhibition of HIV-mediated CD4 T cell depletion by each bNAb treatment was calculated by the following two equations:

*% of inhibition of killing = (% of CD4 T killing in untreated control CD4 T − % of killing in CD4 T treated with bNAbs)/% of CD4 T killing in untreated control CD4 T × 100%*

*% of CD4 T killing in infected sample = CD4 T-cell number in infected sample/CD4 T-cell number in uninfected control × CD8T cell number in uninfected control/CD8 T-cell number in infected sample × 100%*

Because CD8 cells are not killed by HIV, recovered CD8 T-cell numbers were used to normalize the recovered CD4 T-cell numbers for differences arising from cell manipulation from experiment to experiment.

### FACS and gating strategies

Percentage of infection and cell numbers for each sample used in the aforementioned equations was determined by FACS analysis, and gating strategies for each cell subset are shown in Figure S1. FACS staining was performed using a live-dead cell discriminator dye (Zombie aqua) and fluorescently labeled antibodies specific for CD4, CD8, and CD3. Data were collected on an LSR II flow cytometer (BD Biosciences) and analyzed using FlowJo Software (BD Biosciences).

## Data availability

All data are included in the article.

## Supporting information

This article contains supporting information.

## Conflict of interest

The authors declare that they have no conflicts of interest with the contents of this article.
