# Supplementary material for: Bystander CD4 T-cell death is inhibited by broadly neutralizing anti-HIV antibodies only at levels blocking cell-to-cell viral transmission
Source: J Biol Chem. 2021 Aug 19;297(4):101098. doi: 10.1016/j.jbc.2021.101098 (PMC8446805; doi:10.1016/j.jbc.2021.101098)
Supplement: Figure S1 [file mmc1.pdf]

Figure S1 Gating examples of co-culture experiments

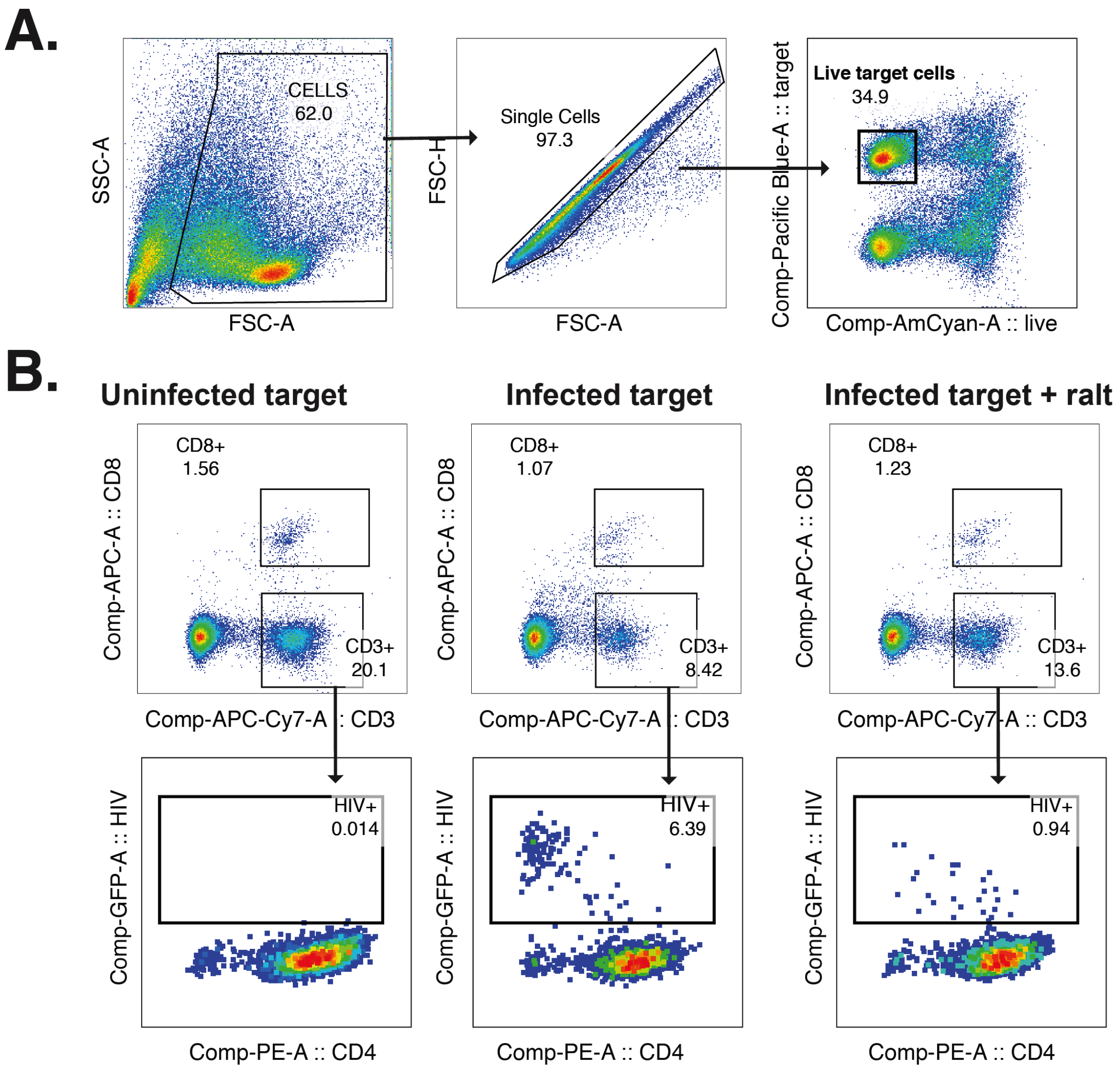

Supplemental Figure 1. Gating strategy for FACS analysis of HIV infection and killing of CD4 T cells.

Fcs files exported from the LSR II flow cytometer were first compensated with single fluorescence-stained beads or cells using FlowJo software. A. Each sample was sequentially pre-gated on intact cells (CELLS) and singlets (Single Cells). In the co-culture experiments, Live target cells were gated as Cell Tracker Blue+ and Zombie aqua -. B. Live target cells were next gated on CD8 T cell (CD8+CD3+), CD4 T cells (CD3+CD8-), and infected CD4 T cells (GFP+ cells in the CD3+CD8- subsets). Statistics including percentage of infection in CD4 T cells, CD4 T cell number, and CD8 T cell number in each sample were exported into FlowJo and further analyzed as described in the Materials and Methods section.
